# Fetal umbilical artery thrombosis: prenatal diagnosis, treatment and follow-up

**DOI:** 10.1186/s13023-022-02563-8

**Published:** 2022-11-12

**Authors:** Xiafang Wu, Chenchen Wei, Ruifeng Chen, Linxian Yang, Weifei Huang, Liang Huang, XinXin Yan, Xuedong Deng, Zhongshan Gou

**Affiliations:** 1 Department of Ultrasonography, Taizhou Hospital of Zhejiang Province, Affiliated to Wenzhou Medical University, Linhai, Zhejiang China; 2 Center for Cardiovascular Disease, The Affiliated Suzhou Hospital of Nanjing Medical University, 242# Guangji Road, Suzhou, 215002 Jiangsu China; 3 Department of Ultrasonography, Affiliated Changsha Central Hospital of South China University, Changsha, Hunan China; 4 Department of Ultrasonography, Tongren People’s Hospital, Tongren, Guizhou, China; 5 Department of Pharmacology, The Affiliated Suzhou Hospital of Nanjing Medical University, 242# Guangji Road, Suzhou, 215002 Jiangsu China; 6 Center for Medical Ultrasound, The Affiliated Suzhou Hospital of Nanjing Medical University, 242# Guangji Road, Suzhou, 215002 Jiangsu China

**Keywords:** Umbilical artery thrombosis, Gestational diabetes mellitus, Umbilical cord abnormalities, Adverse pregnancy outcomes

## Abstract

**Background:**

To analyze the ultrasound imaging and clinical characteristics of fetuses with umbilical artery thrombosis (UAT), explore the potential causes of UAT and construct a prognostic prediction model to guide clinical practice.

**Methods:**

This was a retrospective cohort study of fetal UAT cases examined at two academic tertiary referral care centers from 2014 to 2020. The basic information of the participants was obtained by interview during follow-up, and data on clinical treatment, delivery conditions, diagnosis and confirmation were obtained through medical records. Probable causes of thrombosis were explored by comparative analysis of the UAT group to the control group and by further regression analysis. Multivariable logistic regression models were used to evaluate risk factors for adverse pregnancy outcomes. Receiver operating characteristic (ROC) curves were constructed to evaluate the diagnostic value of the prognostic prediction model.

**Results:**

Thirty fetuses with UAT were included in this study. UAT occurred mostly in the third trimester of pregnancy, and there was an obvious predominance of right UAT. An abnormal pregnancy history (53.3%) was the most common comorbidity, followed by gestational diabetes mellitus (GDM) (20.0%). GDM and umbilical cord (UC) abnormalities were found to be independent risk factors for the development of UAT. After comprehensive decision-making, over two-thirds of the patients with UAT received urgent treatment, and less than one-third received expectant management. Surprisingly, there were no significant differences in fetal outcomes between the urgent treatment and expectant management groups. Multivariate logistic regression analysis showed that gestational age (GA) at clinical diagnosis and UC abnormalities were independent risk factors for adverse pregnancy outcomes (OR 0.781, *p* = 0.042; OR 16.779, *p* = 0.023, respectively). Based on this, we constructed a comprehensive prognostic prediction model. The area under the ROC curve (AUC) was 0.877 (95% CI 0.698–0.970; *p* < 0.001), which suggested that the combination of GA and UC abnormalities was a better predictor for fetal outcomes in our setting.

**Conclusion:**

In summary, maternal GDM and fetal UC abnormalities are independent risk factors for UAT. UAT is more frequently observed on the right side. Moreover, poor clinical outcomes for fetuses with UAT are ascribed mainly to GA and UC abnormalities, which should be comprehensively evaluated to choose the appropriate treatment.

## Background

Umbilical artery thrombosis (UAT) is a rare complication of pregnancy, reportedly affecting approximately 0.025–0.045% of deliveries [1, 2]. Umbilical cord thrombosis, documented by prospective examination of the placenta, occurs in only 1/1300 deliveries, of which UAT accounts for about 30% [2]. However, UAT usually results in a series of adverse pregnancy outcomes, such as intrauterine growth retardation (IUGR), fetal distress, and even intrauterine fetal demise, particularly when UAT occurs during the first half of gestation [1, 2].

Generally, most fetuses with UAT have no specific clinical symptoms or signs. In a few cases, the primary manifestation is a reduction in fetal intrauterine movement, and sometimes, abnormalities, such as persistent fetal bradycardia, are found by fetal heart monitoring [3]. This situation easily leads to a missed diagnosis during conventional obstetric examinations. An insufficient index of suspicion for early diagnosis makes it difficult to provide timely intervention, leading to sudden unexplained intrauterine death.

To the best of our knowledge, only a few case reports or case series have briefly reported the characteristics of fetal UAT. Previous studies have suggested that congenital umbilical cord dysplasia, infections, abnormal maternal blood glucose and the maternal coagulation state is related to the occurrence and development of thromboembolism [1, 4]. However, the etiology and specific mechanism of fetal UAT remain unknown due to the absence of reliable research data.

Here, we present a relatively large sample of fetuses with UAT from two academic tertiary referral care centers examined between 2014 and 2020. The aims of this study were as follows: (1) to share our experience of prenatal diagnosis and intervention approaches for UAT; (2) to explore the risk factors for fetal UAT; and (3) to construct a prognostic prediction model to guide antenatal counseling.

## Methods

### Study population

This was a retrospective cohort study of pregnant women performed in two academic tertiary referral care centers from May 2014 to May 2020. The inclusion criteria were as follows: (1) pregnant women with complete medical histories and laboratory data; (2) pregnant women with fetal UAT confirmation by ultrasound scan; and (3) pregnant women for whom follow-up data regarding the pregnancy outcome from the first detection of fetal UAT to delivery were available. Another thirty-five pregnant women with gestational age (GA)-matched normal fetuses during the same period were randomly selected as the control group. The Research Ethics Committees of the two participating hospitals approved this study, and all pregnant participants signed written informed consent forms.

### Ultrasound examination

*Ultrasound Equipment.* A Voluson™ E8 ultrasound system (GE Healthcare Ultrasound, Milwaukee, WI, USA) coupled with a C1-5-D transducer (frequency 2–5 MHz) was used.

*Routine scanning and assessment.* Fetal GA was determined according to the date of the last menstrual cycle of the mother. For cases in which the date was not available, the fetal GA was estimated according to the combination of the biparietal diameter, head circumference, abdominal circumference and femur length, as measured by ultrasound. Estimated fetal weight (EFW) was calculated using the Hadlock formulas based on selective ultrasonography parameters. Fetal growth restriction was defined as an EFW below the 10th percentile [5, 6].

**Fig. 1:**
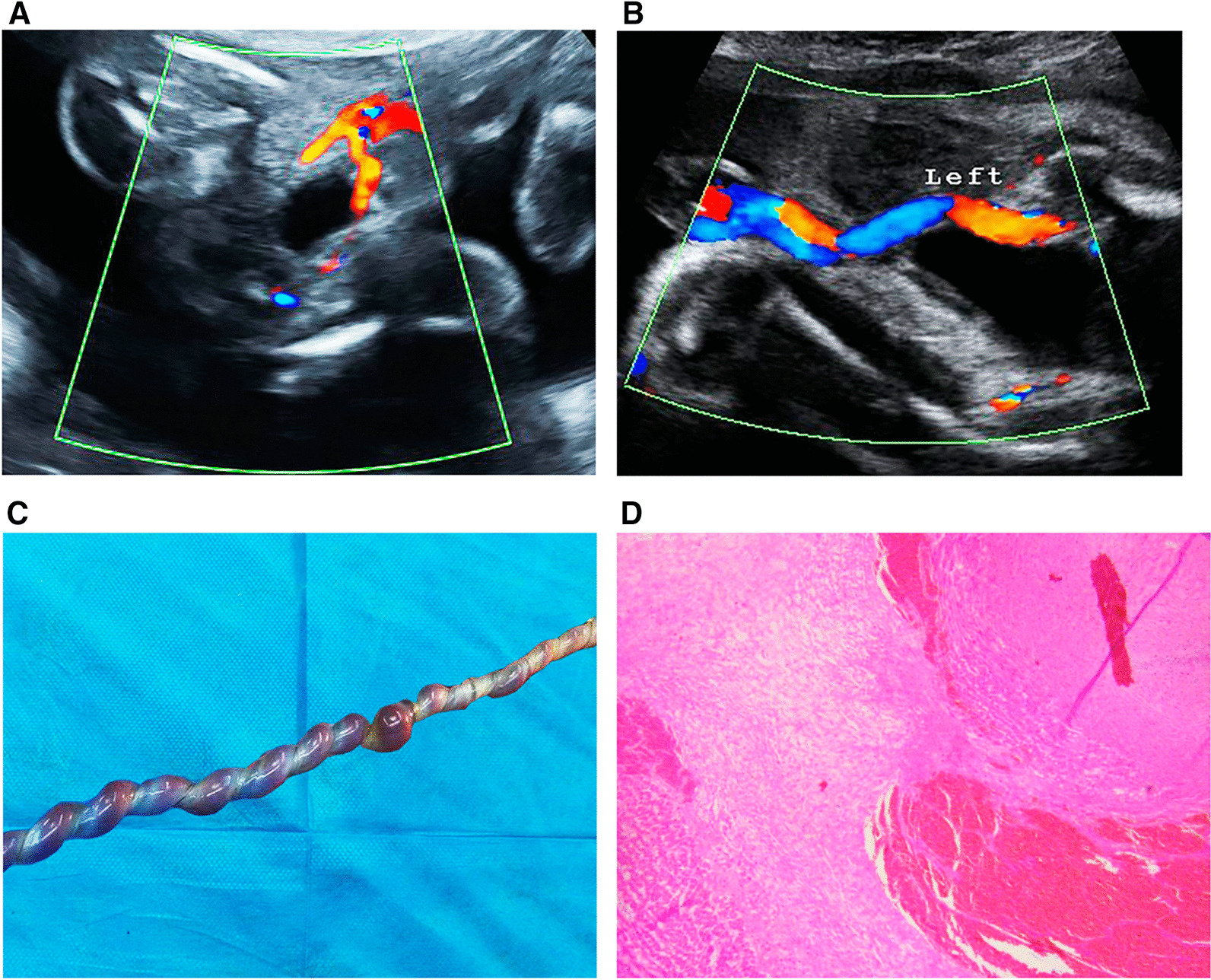
**A** Previous ultrasonic examinations showed bilateral umbilical arteries. **B** One instead of two umbilical arteries was clearly demonstrated later. **C** Gross observation of the umbilical cord. **D** Pathological analysis (HE staining; magnification, × 4 and × 10)

*Identification of fetal UAT.* The prenatal diagnostic protocols for fetal UAT were as follows: (1) previous ultrasonic examinations showed bilateral umbilical arteries (UAs); (2) the combination of two-dimensional ultrasound with color Doppler or power Doppler when necessary showed that one rather than two UAs was clearly demonstrated; and (3) a “ZOOM” of the lumen of the suspected UA revealed that it was filled with hypoechoic materials (e.g., Fig. 1, A and B). In particular, the prenatal diagnosis of fetal UAT was independently made by two highly experienced obstetricians, and if a dispute arose, the final decision was made by a third qualified obstetrician. Once the diagnosis of UAT was confirmed, the hemodynamic situation was carefully assessed according to the fetal cardiovascular profile score [7].

### Prenatal management

For fetuses with a GA less than 32 weeks, we were more inclined to choose protective and expectant management patterns if there were no obvious anomalies of reduced fetal movement and abnormal fetal heart tracing. In contrast, fetuses with a GA greater than 32 weeks were categorized into the urgent treatment group. In the urgent treatment group, emergency cesarean section was performed if vaginal delivery failed. In the expectant management group, home monitoring of the fetal heart rate was conducted once a day, and ultrasound reexamination was performed once a week to reassess the UAT situation and hemodynamic situation. When necessary, the patients were hospitalized. Oxygen inhalation, low-molecular-weight heparin for anticoagulation, ritodrine hydrochloride, magnesium sulfate or other tocolytic agents for fertility preservation were used as appropriate. Dexamethasone was given to mothers to accelerate fetal lung maturation. When fetal intrauterine distress could not be corrected, the fetus was delivered by cesarean section.

### Follow-up

All patients with UAT were followed up closely starting at the first detection of UAT. When the compromised baby was born, the Apgar score was determined immediately. Additionally, data on GA at birth, birth weight and the sex of the baby were recorded. Small for gestational age (SGA) was defined as a birth weight below the 10th percentile for GA based on the Chinese neonatal birth weight curve [8]. Adverse pregnancy outcomes included stillbirth, preterm birth and SGA. The whole course of the UC, especially if there were loops of the UC around the neck and if there were true or false UC knots, was observed and photographed. Then, the whole placenta and UC were sent for further pathological examinations.

### Pathological examination of the UC

The key observations were the appearance, length, coiling index, and true or false knots of the UC. The definitions of UC abnormalities included an excessively long UC (> 80 cm), an excessively short UC (< 40 cm), severe entanglement (more than two cycles of the UC around the neck), true knots, and hypercoiling (coiled cycles per unit length > 90th percentile) [9].

After fixation, the sites of thrombosis in the UAs were embedded in paraffin. Cross-sections were stained with hematoxylin–eosin (HE), and thrombus formation, whether occlusive or nonocclusive, was verified by HE staining. The presence of thrombi was histologically confirmed if agglutinated platelets were covered by condensed fibrin, which contained leukocytes.

### Statistical analysis

Statistical analysis was performed with SPSS 26.0 (IBM Corporation) and MedCalc 20.0.4 (MedCalc Software Ltd, Ostend, Belgium). Comparisons of continuous variables between groups were performed using Student's t-test or the Mann‒Whitney U test, and comparisons of categorical variables were performed using the χ^2^ test. Kaplan‒Meier curves and Cox proportional hazards regression models were used to evaluate the influence of the different interventions on outcomes. We constructed multivariable logistic regression models to explore relevant risk factors. Receiver operating characteristic (ROC) curves were constructed to evaluate the diagnostic value of the parameters. Values were considered statistically significant at *p* < 0.05.

## Results

### Baseline characteristics

In total, 75,708 pregnant women were managed at the two maternity centers during the study period, and of these women, thirty with UAT were included. The prevalence of UAT was approximately 1 in 2524 deliveries. Notably, two fetuses were already found to be dead when UAT was diagnosed during the mothers’ routine obstetric examinations. In both cases, the presence of UAT was confirmed histologically. Approximately 90% of the fetuses were first found to have UAT in the third trimester, and only three fetuses were found to have UAT in the second trimester.

As presented in Table 1, the mean maternal age of the UAT group was 29.5 ± 5.8 years, ranging from 19 to 42 years. Twenty percent of the mothers were of advanced maternal age (> 35 years). However, there was no difference in fetal UAT morbidity between pregnant women aged > 35 years and those aged < 35 years. In the UAT group, an abnormal pregnancy history (53.3%) was the most common comorbidity, followed by gestational diabetes mellitus (GDM, 20.0%) and gestational hypertension (6.7%). When comparing the UAT and control groups, we found differences only in the incidence of maternal GDM (*p* < 0.05).

**Table 1:** The baseline characteristics of the UAT fetuses and comparison to the normal fetuses

	UAT (*n* = 30)	Control (*n* = 35)	*p*
Age (years)	29.53 ± 5.81	29.80 ± 6.15	0.859
*Comorbidities*			
Abnormal pregnancy history	53.3%	42.9%	0.399
GDM	20.0%	2.9%	0.042
GH	6.7%	2.9%	0.591
Multiple pregnancy	3.3%	0.0%	0.462
Nuchal cord	50.0%	34.3%	0.200
Abnormal amniotic fluid	26.7%	28.6%	0.864

*p* value, UAT group *vs.* control group. UAT, umbilical artery thrombosis; GDM, gestational diabetes mellitus; GH, gestational hypertension

### Independent risk factors for fetal UAT

To determine the potential causes of fetal UAT, we took the following risk factors into consideration: maternal age, abnormal pregnancy history, GDM, gestational hypertension, inflammatory response, coagulation function and UC abnormalities. Logistic regression revealed that maternal GDM and fetal UC abnormalities were independent risk factors for UAT (OR 13.286, *p* = 0.022; OR 7.381, *p* = 0.006, respectively) (Table 2).

**Table 2:** Analysis of risk factors for fetal UAT

	Univariate		Multivariate	
	OR (95% CI)	*p*	OR (95% CI)	*p*
Age	0.992 (0.914–1.078)	0.856		
Abnormal pregnancy history	1.524 (0.571–4.065)	0.400		
GDM	8.500 (0.960–75.234)	0.054	13.286 (1.459–120.997)	0.022
GH	2.429 (0.209–28.200)	0.478		
NLR	1.091 (0.902–1.321)	0.369		
FIB	1.209 (0.583–2.507)	0.611		
UC abnormalities	5.333 (1.307–21.757)	0.020	7.381 (1.755–31.038)	0.006
Abnormal amniotic fluid	0.909 (0.305–2.709)	0.864		

*GDM* Gestational diabetes mellitus, *GH* Gestational hypertension, *NLR* Neutrophil to lymphocyte ratio; FIB, Fibrinogen. Umbilical cord (UC) abnormalities: excessive length difference, severe entanglement, true knots and hypercoiling

### Follow-up findings

All participants were followed up to delivery (see Table 3). The majority of the deliveries in the UAT group (72.4%) were performed by cesarean section, while in the control group, the percentage was only 23.5%; the difference was statistically significant (*p* < 0.001).

**Table 3:** Pregnancy outcomes and neonatal evaluations

	UAT (*n* = 30)	Control (*n* = 35)	*p*
*Pattern of delivery*			
Cesarean section	72.4% (21/29)	23.5% (8/34)	0.000
Vaginal delivery	27.6% (8/29)	76.5% (26/34)	
Adverse pregnancy outcomes	50.0%	5.7%	0.000
Stillbirth	13.3%	0.0%	0.040
Preterm birth	33.3%	2.9%	0.001
SGA	6.7%	2.9%	0.591
GA at birth (w)	36.13 ± 3.27	38.63 ± 1.22	0.000
Birth weight (kg)	2.60 ± 0.73	3.20 ± 0.37	0.000
Sex (male)	60.0% (15/25)	54.5% (18/33)	0.678
Apgar score	10.0 (7.5, 10.0)	10.0 (10.0, 10.0)	0.000
Fetal distress	37.9% (11/29)	0.0% (0/34)	0.000

*p* value, UAT group *vs.* control group. Adverse pregnancy outcomes included stillbirth, preterm birth and small for gestational age (SGA). GA, gestational age

During the fetal period, the fetuses in the UAT group were more likely to develop intrauterine distress than those in the control group (37.9% *vs*. 0.0%, *p* < 0.001). Unexpectedly, half of the pregnancies with fetal UAT had adverse pregnancy outcomes, such as stillbirth, preterm birth and SGA. In particular, the prevalence rates of stillbirth and premature birth in the UAT group were significantly higher than those in the control group (*p* < 0.05). At delivery, a smaller GA, lower birth weight, and lower Apgar score were more likely to occur in the UAT group than in the control group (all *p* < 0.001).

*Pathological findings*. All patients in the UAT group were histologically confirmed to have UAT (Fig. 1, C and D), and most of the patients (67.9%) had right UAT.

### The effects of different treatment measures on pregnancy outcomes

Based on the recommendations of the clinicians and the preferences of the pregnant women, the majority of the 30 participants (73.3%) received urgent treatment rather than expectant management. In our comparisons of the clinical characteristics of the patients receiving urgent treatment versus expectant management, we found that the difference in GA at clinical diagnosis was significant (36.19 ± 3.09 *vs*. 28.90 ± 2.92 weeks, *p* < 0.001).

**Fig. 2:**
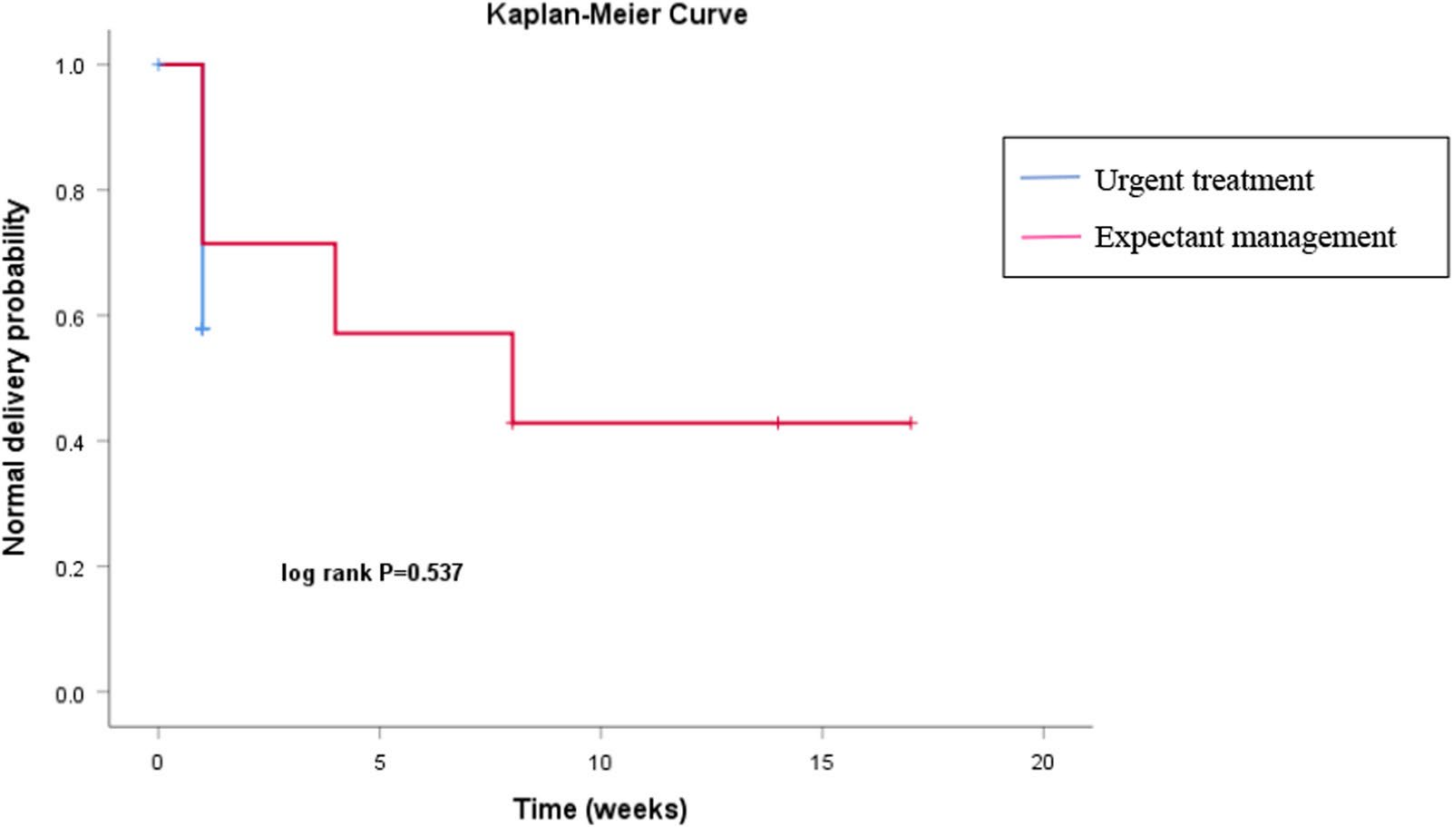
The effects of different treatment measures on pregnancy outcome (urgent treatment *vs*. expectant management)

The Kaplan‒Meier curve indicated that fetal outcomes (with or without adverse pregnancy outcomes) were not significantly affected by whether the pregnancies were managed urgently or expectantly (*p* = 0.537) (Fig. 2). After an adjustment was made for GA as a confounding factor, the different interventions were still not independently associated with adverse pregnancy outcomes among patients with UAT (Table 4).

**Table 4:** Cox regression analysis for the effect of different treatment measures on pregnancy outcomes

	*B*	*S.E*	*Wald*	*P*	*RR*	*95% CI*
GA at clinical diagnosis	− 0.151	0.094	2.570	0.109	0.860	(0.714, 1.034)
Interventions	− 1.514	1.055	2.060	0.151	0.220	(0.028, 1.739)

GA, gestational age

### Prediction for the prognosis of patients with UAT

We performed univariate and multivariable logistic regression analyses to explore risk factors associated with adverse pregnancy outcomes in patients with UAT (Table 5). The regression analysis showed that GA at clinical diagnosis and UC abnormalities were independent predictors for adverse pregnancy outcomes (OR 0.781, *p* = 0.042; OR 16.779, *p* = 0.023, respectively). The smaller the GA at clinical diagnosis was, the greater the possibility of adverse pregnancy outcomes would be.

**Table 5:** Prediction of the risk of adverse pregnancy outcomes

	Univariate		Multivariate	
	OR (95% CI)	*p*	OR (95% CI)	*p*
Age	0.986 (0.869–1.118)	0.822		
Past medical history	1.021 (0.230–4.526)	0.978		
GA at clinical diagnosis	0.779 (0.625–0.971)	0.026	0.781 (0.617–0.991)	0.042
UC abnormalities	16.333 (1.632–163.439)	0.017	16.779 (1.482–190.027)	0.023
Abnormal amniotic fluid	1.222 (0.237–6.315)	0.811		
Fetal growth restriction	1.625 (0.190–13.933)	0.658		
Fetal distress	3.000 (0.571–15.766)	0.194		

*GA* Gestational age, *UC* Umbilical cord

**Fig. 3:**
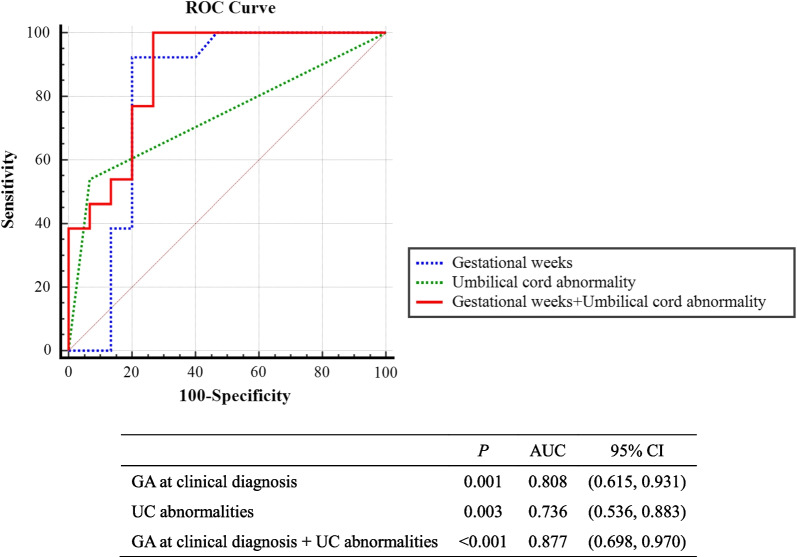
ROC Curve. Receiver operating characteristic (ROC) analysis was used to evaluate the diagnostic value. AUC, area under the curve; GA, gestational age; UC, umbilical cord

The prediction performance of the indices was further analyzed by means of ROC curves based on the results of the regression analysis (Fig. 3). The areas under the ROC curve (AUCs) were 0.808 for GA at clinical diagnosis and 0.736 for UC abnormalities. Importantly, we found that the predictive effect of the combination of GA at clinical diagnosis and UC abnormalities on adverse pregnancy outcomes was better than either risk factor alone, suggesting that the combination of both risk factors may be the best predictive parameter (AUC [95% CI] 0.877 [0.698–0.970]; *p* < 0.001) (*p* < 0.05, combined prediction *vs*. either GA at clinical diagnosis or UC abnormalities). The ROC analysis showed that fetuses with a GA less than 34.8 weeks or the presence of UC abnormalities tended to have adverse pregnancy outcomes (Youden's index was 0.733).

## Discussion

UAT is a rare but potentially dangerous complication. Because of its rarity and limited research data, there is currently no consensus on the treatment strategy for fetal UAT. Additionally, many obstetricians are unfamiliar with UAT. This study reported the initial experience of our centers. The prediction of fetal prognosis and the treatment response underlie important clinical decisions. Notably, our study indicated that the combination of decreased GA and UC abnormalities was of high value for predicting adverse pregnancy outcomes among fetuses with UAT in our setting. No significant differences in adverse outcomes were found between the urgent or expectant management groups.

*Underlying pathogenic factors of fetal UAT.* Our data showed that GDM and UC abnormalities were independent risk factors for the occurrence of UAT. Recent findings in two descriptive case series by Li et al. [10] and Zhu et al. [4] also noted that congenital UC dysplasia and maternal abnormal blood glucose could be likely etiologies. Unfortunately, their data did not provide enough statistical support. GDM was the most frequent concomitant disease (20%) found to be associated with UAT. The frequency of GDM in this study was far higher than that in the general pregnant population [11]. Previous case series have also reported that some pregnant women with fetal UAT had GDM [12, 13]. Brown et al. (2019) demonstrated a correlation between maternal diabetes and fetal thrombotic vasculopathy [14]. Although the cause was unknown, it may be explained using Virchow’s hypothesis for thrombosis (blood stasis, endothelial injury, and hypercoagulability) [15]. The UC, which is a cord-like structure connecting the fetal umbilicus and the placenta, is an important channel for nutrient metabolism and material exchange between a fetus and its mother. An abnormal UC anatomy or mechanical injury to the UC, including excessive length, twisting, true knots, hypercoiling, and compression, may be associated with blood flow restriction and the occurrence of umbilical vessel thrombosis [16–19]. Most UAT cases reported previously were related to UC abnormalities (e.g., Oliveira et al.; Wei et al. [19, 20]). In our study, notable UC abnormalities were present in a large proportion (30.0%) of fetuses with UAT. Umbilical vascular thrombosis may also be linked to other common risk factors, such as abnormal coagulation function and infection [12]. However, consistent with the study by Wei et al., we did not find obvious abnormalities in maternal inflammation or coagulation [20].

*Prenatal diagnosis.* The prenatal diagnosis of UAT depends mainly on ultrasonography, and UAT is easily detected by color or power Doppler flow tests. Abnormal Doppler waveforms of umbilical vessels may be detected before obvious anomalies of fetal circulation occur [21]. Cook et al. first proposed that prenatal early diagnosis of UAT could be made by ultrasound examination, after which some scholars described it as the "orange grabbed" sign [22, 23]. Our study had similar findings. It is important to maintain high vigilance in high-risk pregnancies, especially those with GDM and UC abnormalities. Interestingly, the left UA was more commonly absent in fetuses with a single umbilical artery, but in fetuses with UAT, the right UA was more frequently involved [24]. Further research is needed to explore these differences and complexities.

*Prognosis*. Here, we propose a predictive model based on GA and UC abnormalities for evaluating pregnancy outcomes. In this model, fetuses with a smaller GA or structural abnormalities in the UC are more likely to have adverse pregnancy outcomes. For patients hospitalized due to disease, a lower GA tends to be indicative of a poor prognosis [25]. A hospital-based decade-long retrospective study in Taiwan found that umbilical cord pathology, including stricture, true knots, strangulation of the fetus, and prolapse, was the most common cause of third-trimester intrauterine fetal demise [26]. In the present study, among the UCs of the confirmed UAT patients, six patients had hypercoiling, five had severe entanglement, and one had excessive length. Clearly, excessive length would increase the risk of UC entanglement. Moreover, fetuses with an excessive UC length, a non-reassuring sign, have significantly increased rates of respiratory distress and perinatal death than those without this sign [27, 28]. Many studies have emphasized the correlation between abnormal umbilical coiling and adverse perinatal outcomes. A hypercoiled UC was significantly associated with thrombosis in umbilical vessels, preterm delivery, aneuploidy and fetal anomalies, increasing the risk of fetal death [29, 30]. Ernst et al. also suggested that hypercoiled UCs with certain gross patterns might be associated with chronic fetal vascular obstruction and stillbirth [31]. Obstetricians should comprehensively assess GA and whether excessive length, severe entanglement, or hypercoiling exists in the UC by ultrasound. This will help obstetricians better grasp the progression and alleviate the fear and anxiety of pregnant women.

*Interventions*. Obstetricians might be hesitant to intervene when managing a fetus with UAT. Obstetricians often make treatment choices according to the fetal status and their own understanding and experience [3, 13]. As mentioned in the Methods section, we are sharing our experience with UAT over several years. Fortunately, after implementation of the current screening and therapeutic strategy, the expectant management group did not have worse fetal outcomes than the urgent treatment group. Therefore, the possibility of fetal protection should be considered in fetuses with a very low GA, while urgent delivery is suggested for fetuses with a higher GA to avert unnecessary fetal loss. In addition, combined with the above prognostic predictors, it is suggested that fetuses with obvious UC abnormalities be treated aggressively.

The strengths of this study are the relatively large sample size of patients with fetal UAT, the completeness of the follow-up and the real-world nature of the data. However, the present study has several potential limitations. The major limitation is that the current evidence is based on retrospective studies and registry data, and it is unknown whether our results can be generalized to the broader population with UAT.

## Conclusions

In summary, the current study reveals the following: (1) maternal GDM and fetal UC abnormalities are two independent risk factors for UAT, and close ultrasound monitoring is suggested to improve the early detection of UAT among these high-risk pregnancies; (2) UAT more frequently appears on the right side; and (3) adverse pregnancy outcomes are closely attributed to GA and the condition of the UC, which should be the main consideration when obstetricians decide whether to provide urgent treatment or expectant management.

## Data Availability

The datasets used during the current study are available from the corresponding author on reasonable request.

## Abbreviations

EFW: Estimated fetal weight
GA: Gestational age
GDM: Gestational diabetes mellitus
IUGR: Intrauterine growth retardation
ROC: Receiver operating characteristic
SGA: Small for gestational age
UA: Umbilical arteries
UAT: Umbilical artery thrombosis
UC: Umbilical cord
